# Supplementary material for: Impact of Early Weight Catch-Up on 6-Year Neurodevelopment and Overweight/Obesity in Children Born Small-for-Gestational-Age
Source: Children (Basel). 2025 Dec 31;13(1):69. doi: 10.3390/children13010069 (PMC12840361; doi:10.3390/children13010069)
Supplement: Supplementary file 1 [file children-13-00069-s001.zip › children-3996803-supplementary.pdf]

List of online supplemental files

Checklist of Recommendations for Reporting of Observational Studies Using the Reporting of Studies Conducted Using Observational Routinely Collected Health Data (RECORD) Guidelines

Supplemental Table S1. Validation data of K-DST

Supplemental Table S2. List of items in the seventh round of the Korean Developmental Screening Test for Infants and Children

Supplemental Table S3. Model-based predicted probabilities of obesity and suboptimal K-DST outcomes at age 6 according to weight-for-age z-score at age 2

Checklist of Recommendations for Reporting of Observational Studies Using the Reporting of Studies Conducted Using Observational Routinely Collected Health Data (RECORD) Guidelines

	Item No	Recommendation	Reported
Title and abstract	1	(a) Indicate the study's design with a commonly used term in the title or the abstract	Page 1,2
		(b) Provide in the abstract an informative and balanced summary of what was done and what was found	Page 1,2
**Introduction**			
Background /rationale	2	Explain the scientific background and rationale for the investigation being reported	Page 3,4
Objectives	3	State specific objectives, including any prespecified hypotheses	Page 3,4
**Methods**			
Study design	4	Present key elements of study design early in the paper	Page 3
Setting	5	Describe the setting, locations, and relevant dates, including periods of recruitment, exposure, follow-up, and data collection	Page 3
Participants	6	(a) Give the eligibility criteria, and the sources and methods of selection of participants. Describe methods of follow-up	Page 3
		(b) For matched studies, give matching criteria and number of exposed and unexposed	Page 3,4
Variables	7	Clearly define all outcomes, exposures, predictors, potential confounders, and effect modifiers. Give diagnostic criteria, if applicable	Page 4
Data sources /measurement	8	For each variable of interest, give sources of data and details of methods of assessment (measurement). Describe comparability of assessment methods if there is more than one group	Page 3,4
Bias	9	Describe any efforts to address potential sources of bias	Page 4
Study size	10	Explain how the study size was arrived at	Page 3
Quantitative variables	11	Explain how quantitative variables were handled in the analyses. If applicable, describe which groupings were chosen and why	Page 4,5
Statistical methods	12	(a) Describe all statistical methods, including those used to control for confounding	Page 5
		(b) Describe any methods used to examine subgroups and interactions	Page 5
		(c) Explain how missing data were addressed	Page 5
		(d) If applicable, explain how loss to follow-up was addressed	Page 5
		(e) Describe any sensitivity analyses	Page 5
**Results**			
Participants	13	(a) Report numbers of individuals at each stage of study, e.g., numbers potentially eligible, examined for eligibility, confirmed eligible, included in the study, completing follow-up, and analyzed	Page 4
		(b) Give reasons for non-participation at each stage	Page 4
		(c) Consider use of a flow diagram	Page 4
Descriptive data	14	(a) Give characteristics of study participants (e.g. demographic, clinical, social) and information on exposures and potential confounders	Page 5,6

		(b) Indicate number of participants with missing data for each variable of interest	Page 5,6
		(c) Summarize follow-up time (e.g. average and total amount)	Page 5,6
Outcome data	15	Report numbers of outcome events or summary measures over time	Page 7-11
Main results	16	(a) Give unadjusted estimates and, if applicable, confounder-adjusted estimates and their precision (e.g., 95% confidence interval). Make clear which confounders were adjusted for and why they were included	Page 7-11
		(b) Report category boundaries when continuous variables were categorized	Page 7-11
		(c) If relevant, consider translating estimates of relative risk into absolute risk for a meaningful time period	Page 7-11
Other analyses	17	Report other analyses done, e.g., analyses of subgroups and interactions, and sensitivity analyses	Page 7-11
**Discussion**			
Key result	18	Summarize key results with reference to study objectives	Page 11-13
Limitation	19	Discuss limitations of the study, taking into account sources of potential bias or imprecision. Discuss both direction and magnitude of any potential bias	Page 11-13
Interpretation	20	Give a cautious overall interpretation of results considering objectives, limitations, multiplicity of analyses, results from similar studies, and other relevant evidence	Page 11-13
Generalizability	21	Discuss the generalizability (external validity) of the study results	Page 11-13
Other information			
Funding	22	Give the source of funding and the role of the funders for the present study and, if applicable, for the original study on which the present article is based	Page 13

Supplemental Table S1. Validation data of K-DST

Chung et al. (2020) [1]	Internal consistency of developmental domains of the K-DST: Cronbach alpha, 0.84 – 0.88 of all domains at 66 – 71 months of age<br>Sensitivity, 0.886; Specificity, 0.951; Accuracy, 0.921; False-positive, 4.9%; False-negative, 11.4%.<br>AUC, cerebral palsy, 0.971 – 0.936; developmental language disorder, 0.924 – 0.957; autism spectrum disorder, 0.999; intellectual disability, 0.951 – 0.964.<br>Correlation between K-DST and K-BSID-II, *r* = 0.38 – 0.68.<br>Correlation between K-DST and K-WPPSI, *r* = 0.48 – 0.74.
Jang (2019) [2]	Sensitivity, 82.9%; specificity, 90.9%; PPV, 98.1%, NPV 48.8%, Accuracy 84.1%<br>Correlation between K-DST and K-BSID-II, *r* = 0.336 – 0.478.<br>Correlation between K-DST and K-WPPSI-III, *r* = 0.216 – 0.658.

Abbreviations: K-DST, Korean Developmental Screening Test for Infants and Children; AUC, area under the curve; K-BSID-II, Korean Bayley Scales of Infant Development-II; K-WPPSI, Korean Wechsler Preschool and Primary Scales of Intelligence

Supplemental Table S2. List of items in the seventh round of the Korean Developmental Screening Test for Infants and Children

Gross motor skills	1. Your child can stop a rolling ball with her or his feet.<br>2. If you throw a tennis ball-sized ball from a distance of 2 m, your child can catch it with both hands.<br>3. Your child can bounce a ball on the floor once.<br>4. Your child can jump over a rope at a height just below the knees.<br>5. Your child can jump alternately with one foot.<br>6. Your child can do at least one jump on a jump rope.<br>7. Your child can lift one leg and hold it with two hands, and run more than three steps.<br>8. Your child can kick a rolling ball.
Fine motor skills	1. Your child can touch his or her thumb to the other four fingers one after the other.<br>2. Your child can draw a triangle if you show her/him a triangle.<br>3. Your child can cut a circle with scissors.<br>4. Your child can draw the shape of a simple car.<br>5. Your child can pour water from a kettle or water bottle into a cup with little to no spillage.<br>6. Your child can draw a diamond if you show her/him a diamond.<br>7. Your child can draw objects, such as a house, a tree, and an animal, in a sufficiently recognizable form.<br>8. Your child can tie a ribbon.
Cognition	1. Your child can distinguish her/his left from right.<br>2. Your child can recite the days of the week in order.<br>3. Your child knows that 500 won coins are worth more than 100 won coins.<br>4. Your child can say her/his birthday.<br>5. Your child can write down numbers from 11 to 20.<br>6. Your child can subtract single-digit numbers (e.g., 5 – 2 = 3).<br>7. Your child can remember the phone number of her/his mother, father, or guardian.<br>8. Your child can accurately point to today's date (month and day) on the calendar.
Language	1. Your child knows the opposite of familiar words (e.g., hot and cold, big and small).<br>2. Your child can recognize the meaning of simple jokes or colloquialism.<br>3. When you ask your child what a word means, she/he can explain the meaning of the word (e.g., When you ask, "What are shoes?," your child can answer, "What I wear when I go outside.").<br>4. Your child can answer questions about hypothetical situations, such as "What would happen if ~?" (e.g., "What if you have a younger brother?").<br>5. Your child can play word chain games.<br>6. Your child can write her/his name or two or four words without looking at a reference (e.g., traffic lights, South Korea).<br>7. Your child can tell a simple joke.<br>8. Your child can understand and recite simple proverbs.
Sociality	1. Your child can easily get along with a person he/she meets for the first time.<br>2. Your child can play rule-based games with her/his peers.<br>3. Your child can talk about her/his thoughts and listen to her/his friends.<br>4. Your child can talk to her/his friends about how to play a game.<br>5. Your child can actively get along with her/his friends.<br>6. Your child can voluntarily say "hello" to someone she/he knows.<br>7. Your child often invites friends over to her/his house to play.<br>8. Your child often calls friends and family.
Self-care	1. Your child can use a spoon to spread butter or jam on bread.<br>2. Your child can wipe herself/himself with a towel after taking a bath.<br>3. Your child can zip up a shirt by herself/himself.<br>4. Your child can change clothes on her/his own if the clothes are dirty.<br>5. Your child can open a milk carton by herself/himself.

	6. Your child can undertake one household chore for a period of time (a week or so) (e.g., placing cutlery, organizing toys, etc.).<br>7. Your child can bathe by herself/himself, except for washing her/his hair.<br>8. Your child can clean her or his own anal area after having a bowel movement.

Supplemental Table S3. Model-based predicted probabilities of obesity and suboptimal K-DST outcomes at age 6 according to weight-for-age Z score at age 2

Weight-for-age Z-score at age 2	Predicted probability of obesity at age 6	Predicted probability of suboptimal K-DST results at age 6	Weight-for-age Z-score at age 2	Predicted probability of obesity at age 6	Predicted probability of suboptimal K-DST results at age 6
-3	0.000	0.127	0	0.041	0.022
-2.9	0.000	0.12	0.1	0.046	0.022
-2.8	0.001	0.114	0.2	0.052	0.022
-2.7	0.001	0.107	0.3	0.059	0.022
-2.6	0.002	0.1	0.4	0.066	0.022
-2.5	0.002	0.094	0.5	0.074	0.023
-2.4	0.002	0.087	0.6	0.084	0.023
-2.3	0.003	0.081	0.7	0.093	0.024
-2.2	0.003	0.074	0.8	0.103	0.024
-2.1	0.004	0.068	0.9	0.114	0.025
-2	0.004	0.062	1	0.130	0.026
-1.9	0.005	0.056	1.1	0.145	0.027
-1.8	0.005	0.051	1.2	0.160	0.028
-1.7	0.006	0.045	1.3	0.175	0.028
-1.6	0.007	0.041	1.4	0.190	0.029
-1.5	0.007	0.038	1.5	0.206	0.03
-1.4	0.008	0.036	1.6	0.221	0.031
-1.3	0.009	0.034	1.7	0.236	0.032
-1.2	0.010	0.032	1.8	0.251	0.033
-1.1	0.011	0.03	1.9	0.266	0.034
-1	0.013	0.029	2	0.282	0.034
-0.9	0.014	0.027	2.1	0.297	0.035
-0.8	0.016	0.026	2.2	0.312	0.036
-0.7	0.018	0.025	2.3	0.327	0.037
-0.6	0.020	0.024	2.4	0.343	0.038
-0.5	0.023	0.024	2.5	0.358	0.039
-0.4	0.025	0.023	2.6	0.373	0.04
-0.3	0.029	0.023	2.7	0.388	0.04
-0.2	0.032	0.022	2.8	0.403	0.041
-0.1	0.036	0.022	2.9	0.419	0.042
			3	0.434	0.043

Reference

[1] Chung HJ, Yang D, Kim GH, et al. Development of the Korean Developmental Screening Test for Infants and Children (K-DST). *Clin Exp Pediatr*. Nov 2020;63(11):438-446. doi:10.3345/cep.2020.00640
[2] Jang CH, Kim SW, Jeon HR, et al. Clinical Usefulness of the Korean Developmental Screening Test (K-DST) for Developmental Delays. *Ann Rehabil Med*. Aug 2019;43(4):490-496. doi:10.5535/arm.2019.43.4.490
